# Health system-related barriers to prenatal care management in low- and middle-income countries: a systematic review of the qualitative literature

**DOI:** 10.1017/S1463423622000706

**Published:** 2023-02-27

**Authors:** Mohammad Mohseni, Haleh Mousavi Isfahani, Ahmad Moosavi, Elham Dehghanpour Mohammadian, Fatemeh Mirmohammadi, Fatemeh Ghazanfari, Shiler Ahmadi

**Affiliations:** 1 Health Management and Economics Research Center, Isfahan University of Medical Sciences, Isfahan, Iran; 2 School of Health Management and Information Sciences, Iran University of Medical Sciences, Tehran, Iran; 3 Department of Health and Community Medicine, Dezful University of Medical Sciences, Dezful, Iran; 4 Department of Midwifery, Faculty of Nursing and Midwifery, Islamic Azad University, Zanjan Branch, Zanjan, Iran; 5 Director of Fatima Midwifery Services & Counseling Clinic, Tehran, Iran; 6 Department of Health Management and Economics, School of Public Health, Tehran University of Medical Sciences, Tehran, Iran; 7 Department of Nursing and Midwifery, Sanandaj Branch, Islamic Azad University, Sanandaj, Iran

**Keywords:** low- and middle-income countries, prenatal care, qualitative research, systematic review

## Abstract

**Background:**

Appropriate prenatal care (PNC) is essential for improving maternal and infant health; nevertheless, millions of women in low- and middle-income countries (LMICs) do not receive it properly. The objective of this review is to identify and summarize the qualitative studies that report on health system-related barriers in PNC management in LMICs.

**Methods:**

This systematic review was conducted in 2022. A range of electronic databases including PubMed, Web of Knowledge, CINHAL, SCOPUS, Embase, and Science Direct were searched for qualitative studies conducted in LMICs. The reference lists of eligible studies also were hand searched. The studies that reported health system-related barrier of PNC management from the perspectives of PNC stakeholders were considered for inclusion. Study quality assessment was performed applying the Critical Appraisal Skills Programme (CASP) checklist, and thematic analyses performed.

**Results:**

Of the 32 included studies, 25 (78%) were published either in or after 2013. The total population sample included 1677 participants including 629 pregnant women, 122 mothers, 240 healthcare providers, 54 key informed, 164 women of childbearing age, 380 community members, and 88 participants from other groups (such as male partners and relatives). Of 32 studies meeting inclusion criteria, four major themes emerged: (1) healthcare provider-related issues; (2) service delivery issues; (3) inaccessible PNC; and (4) poor PNC infrastructure.

**Conclusion:**

This systematic review provided essential findings regarding PNC barriers in LMICs to help inform the development of effective PNC strategies and public policy programs.

## Background

There has been widespread and continuing concern about maternal and newborn health across the world (United Nations, 2021; Chowdhury *et al.*, 2022). Globally, around half a million women die as a result of pregnancy and birth complications each year (Hadden, 2012). In 2017, approximately 810 pregnant women died every day from preventable pregnancy- and childbirth-related causes (World Health Organization, 2019). In addition, it is estimated that about 15 million babies (1 in 10) are born prematurely each year around the world, over one million of them die soon after birth, and a considerable number of the remaining ones experience several lifelong disabilities (Adane *et al.*, 2014). These statistics raise challenges for healthcare authorities and professionals to improve maternal and child health.

There is a wide agreement that early and adequate prenatal care (PNC) is essential to improve maternal and child health (Krukowski *et al.*, 2022; Racine *et al.*, 2022). PNC, also known as antenatal care, is a routine preventive healthcare service, with the potential to improve healthy development of a child and to decrease maternal mortality by allowing early identification and treatment of potential pregnancy-related complications, treating medical conditions, and promoting healthier lifestyle (Heaman *et al.*, 2007; 2014). PNC, together with postpartum care, was recognized as an essential strategy to achieve targets such as reduced child mortality as part of Millennium Development Goals 4 and 5 (United Nations, 2008). World Health Organization (WHO) recommends that pregnant women should have at least four PNC appointments during their pregnancy, with supplementary appointments if they experience any complications (World Health Organization, 2021).

Despite the well-documented advantages of PNC services, many women globally do not receive appropriate PNC (Fagbamigbe & Idemudia, 2015). This issue is more highlighted in low- and middle-income countries (LMICs), in which at least 94% of all maternal deaths occur and most of them could have been prevented. For example, according to WHO reports, only 39% of women meet the target of four or more PNC appointments (World Health Organization, 2021). This is while pregnancy- and childbirth-related complications are the leading cause of fatality and disability among women of reproductible age in LMICs (Azmat *et al.*, 2021).

Identifying major barriers to PNC services is important for designing and implementing strategies to improve maternal and child health. In this regard, qualitative studies may provide fresh insights into pertinent issues in specific settings of LMICs. Systematic review and synthesis of qualitative studies can systematically gather relevant evidences regarding PNC barrier in LMICs. While several systematic reviews have been published on PNC utilizations, no systematic review yet has been conducted to comprehensively evaluate the health system-related barriers to PNC in LMICs. Most of the previous reviews focused merely on a single aspect of PNC, such as healthcare-seeking behaviors (Lassi *et al.*, 2019), or among only a specific population, such as those who received inadequate PNC (Finlayson & Downe, 2013; Cisse *et al.*, 2022). Thus, the aim of this review was to systematically identify and summarize qualitative studies to describe the healthcare system-related barriers to all dimensions of PNC (such as utilization and attendance barriers, late initiation, or poor quality of care) from all stakeholders’ perspective (including pregnant women, healthcare providers, and community members) in LMICs.

## Methods

A systematic review was carried out following the Preferred Reporting Items for Systematic Reviews and Meta-Analyses (Page *et al.*, 2021). A systematic search of six electronic databases was undertaken to identify studies focusing on healthcare system-related barriers for PNC in LMICs. The following electronic databases were searched: PubMed, Web of Knowledge, CINHAL, SCOPUS, Embase, and Science Direct. Databases were searched from inception to July 24, 2022 (final search). The search strategy comprises three components, with terms including (i) PNC, (ii) LMICs, and (iii) qualitative studies (Table 1) with a combination of Medical Subject Headings and free text (Title/Abstract).

**Table 1.** Search strategy elements

Prenatal care	(Antenatal care or antenatal service? or antenatal support or prenatal care or prenatal service? or prenatal support or antepartum care or antepartum service? or antepartum support or perinatal care or perinatal service? or perinatal support or maternal care or maternal service? or maternal healthcare or maternal healthcare or maternal support or pregnancy care or pregnancy service? or pregnancy support). ti, ab, kw.
Qualitative studies	Interview/.exp or qualitative research/.exp or (qualitative study or qualitative research or focus-group* or experience* or attitude): ti, ab.
LMICs	(Afghanistan or Albania or Algeria American Samoa or Angola or Argentina or Armenia or Azerbaijan or Bangladesh or Belarus or Belize or Benin or Bhutan Bolivia or Bosnia and Herzegovina or Botswana or Brazil or Bulgaria or Burkina Faso or Burundi or Cabo Verde or Cambodia or Cameroon or Central Africa* or Chad or China or Colombia or Comoros or Congo or Costa Rica or Cote D’Ivoire or Cuba or Djibouti or Dominica or Dominican or Ecuador or Egypt or El Salvador or Guinea or Eritrea or Eswatini or Ethiopia or Fiji or Gabon or Gambia or Georgia or Ghana or Grenada or Guatemala or Guinea or Guinea-Bissau or Guyana or Haiti or Honduras or India or Indonesia or Iran or Iraq or Jamaica or Jordan or Kazakhstan or Kenya or Kiribati or Korea people’s rep* or North Korea or Kosovo or Kyrgyz or Lao or Lebanon or Lesotho or Liberia or Libya or Madagascar or Malawi or Malaysia or Maldives or Mali or Marshall Islands or Mauritania or Mauritius or Mexico or Micronesia or Moldova or Mongolia or Montenegro or morocco or Mozambique or Myanmar or Namibia or Nauru or Nepal or Nicaragua or Niger or Nigeria or North Macedonia or Pakistan or Papua New Guinea or Paraguay or Peru or Philippines or Romania* or Russia* or Rwanda or Samoa or Sao Tome Principe or Senegal or Serbia or sierra Leone or Solomon islands or Somalia or south Africa or Sri Lanka or Lucia or Vincent the Grenadines or Sudan or Suriname or Syria* or Tajik* or Tanzania or Thailand or Timor-Leste or Togo or Tonga or Tunisia or turkey or Turkmen* or Tuvalu or Uganda or Ukraine or Uzbek* or Vanuatu or Venezuela or Vietnam or west bank or Gaza or Yemen or Zambia or Zimbabwe).hw,kf,ti,ab,cp.

Terms recommended by McMaster University Health Information Research Unit were selected as “qualitative study” filters (Mcmaster University, 2016). Search terms connected with Boolean operators “AND” and “OR”. In addition to the electronic database search, the reference lists of included studies were also reviewed for additional relevant studies. The retrieved records were handled using Endnote V.8.

### Inclusion/exclusion criteria

All studies with an aim to qualitatively identify and report on pregnant women’s or/and any healthcare providers’ or general population’s views of health system-related barriers to PNC were eligible. For the purpose of this review, we defied healthcare system as a set of activities and actors whose primary objective is to improve population health through provision of public or private medical services (Panda & Thakur, 2016). Thus, we considered studies that concerned with health system inputs (e.g., physical or human resources) and characteristics (e.g., deliver, financing, and governence). We defined LMICs according to World Bank criteria. We did not apply any participant’s age/sex restrictions during the search. Mixed-methods studies from which it was possible to extract relevant findings derived from qualitative research were also included. We considered any domains of PNC (e.g., utilization barriers, delay in PNC utilization, provision of PNC, and quality of PNC).

We excluded studies focusing only on special pregnant groups such as HIV-infected women. We also excluded studies that did not identify or discuss the health system related, that is, we excluded studies that focused on factors other than health system-related factors such as family culture. Studies that were not peer reviewed, such as dissertations, were also excluded. We excluded unpublished gray literature because of the fact that they score poorly on methodological quality.

Studies not focusing on PNC or focused on specific PNC initiatives such as group antenatal care were also excluded. Moreover, papers not focusing on LMICs were excluded. We also excluded survey-based studies with close-ended questions. In addition, articles of non-English publications were not included in this review as there was no funding for translation.

### Study selection

Results of search strategy were imported to an EndNote library, and it was shared between the two reviewers after removing the duplicates. These two reviewers independently conducted the screening of the titles and abstracts against inclusion and exclusion criteria. This process was followed by obtaining full texts and double screening of potentially eligible studies. Discrepancies regarding eligibility were handled by discussion among team members.

### Quality assessment and data extraction

All articles remaining after full-text verification were quality assessed in terms of study design and other characteristics using Critical Appraisal Skills Programme (CASP) tool (CASP UK, 2018). Quality appraisal was done independently by two authors, and any disagreements were solved by discussion. All studies were included regardless of quality appraisal results. We performed extraction of data based on the main review question: healthcare system-related barriers to PNC. Two reviewers extracted independently this data from the included studies, and disagreements were resolved through discussion. Data extracted using a customized data extraction form piloted on three studies. Data were extracted from each paper on first author, publication year, country, participants, data collection method, and key relevant findings.

### Data synthesis

As a qualitative evidence synthesis method, we applied thematic synthesis (Thomas & Harden, 2008), which has been recognized as a routine approach in the synthesis of qualitative research in systematic reviews (Joseph *et al.*, 2019; Dattilo *et al.*, 2020). This technique is designed to identify new themes, while preserving an explicit and transparent link between conclusions and the text of primary studies. Synthesis included becoming familiar with the data by open-minded reading of each study and being familiar with the results, line-by-line coding of each study results, and categorization of codes into groups of health system-related barriers to PNC. This data synthesis process was conducted by two reviewers.

## Results

**Figure 1.**
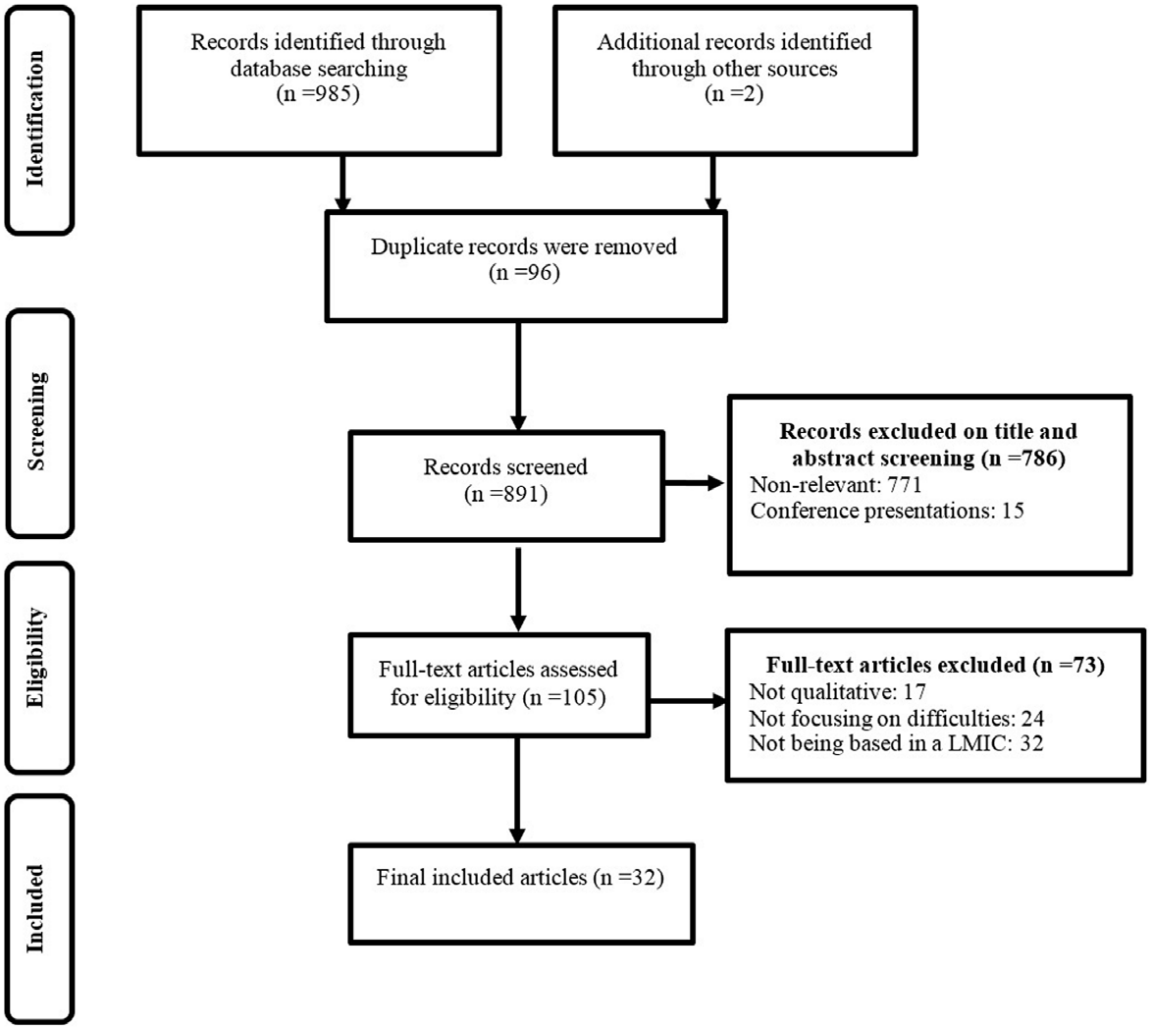
Flow diagram for study selection

The defined search strategy identified 987 citations, of which 96 articles were removed due to duplication while 891 potentially relevant studies were retained for further screening. Screening of titles and abstracts of remaining articles for their eligibility resulted in exclusion of 786 obviously irrelevant records. In the next step, the full text of the remaining 105 studies was assessed for eligibility. During this phase, 73 studies were excluded from the review because of meting exclusion criteria. The remaining 32 studies were critically appraised and included in the review (Table 2). A flow diagram of the study selection process is provided in Figure 1.

**Table 2.** Overview of included studies

First author /year	Country	Objectives	Data collection	participants	Analysis approach	Key emerged themes	CASP score
Udenigwe (Udenigwe *et al*., 2021)	Nigeria	Perspectives of policymakers and health workers on facilitators and barriers to women’s use of skilled pregnancy care	In-depth interviews	13 key stakeholders (policymakers and healthcare providers	Thematic analysis	* Financial constraints * Women’s lack of decision-making power * Ignorance and poor understanding of health * Competitive services offered by traditional birth attendants * Previous negative experience with skilled healthcare * Shortage of health workforce * Poor financing * Governance of the health system	8
Hajian (Hajian *et al.*, 2022)	Iran	Explore the barriers and facilitators of Iranian men’s involvement in perinatal care	In-depth interviews	21 Pregnant women, spouses, policymakers, and midwifery service providers	Content analysis	* Individual factors * Organizational factors * Organizational factors * Legislative factors	8
Mourtada, 2021 (Mourtada *et al*., 2021)	Syria	Compares two governorates to highlight the barriers to women’s adequate uptake of ANC that existed in Syria preconflict	Semi-structured interviews	30 pregnant women, 15 observation sessions at health facilities	Framework analysis	* Women’s assessment of their health status and reasoning of causes of ill health in pregnancy * Women’s evaluation of the risks of seeking ANC * Women’s appraisal of the value of different types of service providers.	7
Tsegaye, 2021(Tsegaye *et al*., 2021)	Ethiopia	Potential contributing barriers to loss to follow-up of pregnant women from antenatal care services in villages	In-depth interviews	20 zonal, woreda and health center managers, midwives and health extension workers	Thematic analysis	* Shortage of the required medical equipment, drugs, and other supplies * Poor care, respect, and receptiveness of service providers * Lack and cost of transport * Community culture and pervious maternal experiences * maternal sociodemographic factors like maternal age and educational status	7
Dadras, 2020 (Dadras *et al.*, 2020)	Iran	Explores the potential barriers to prenatal care among Afghan women in Iran.	Face-to-face interviews	30 pregnant Afghan women	Content analysis	* The financial constraints * lack of affordable health insurance with adequate coverage of prenatal care services	8
Akter, 2018 (Akter *et al.*, 2018)	Bangladesh	To explore perceived barriers to prenatal care among pregnant women	A descriptive qualitative research	20 pregnant women and 20 of their significant others	Content analysis	* a lack of female doctors * unaffordable laboratory tests and medications	9
Alanazy, 2019 (Alanazy *et al*., 2019)	Saudi Arabia	To understand the beliefs of pregnant women and health professionals about the factors leading to low attendance rates	A qualitative exploratory study	antenatal (*n* = 14) and postnatal women (*n* = 7) and health professionals working with pregnant and new mothers (*n* = 9)	Thematic analysis	* poor, or a lack of facilities * Long waiting time * Lack of specialized facilities * Dismissive staff * Pressurising staff * Poor care	9
Andrew EV, 2014 (Andrew *et al.*, 2014)	Papua New Guinea	To explore the influences on ANC attendance and timing of first visit	Free listing and sorting of terms and definitions, focus group discussions, in-depth interviews, observation	Pregnant women (*n* = 9), their relatives (*n* = 13), biomedical and traditional health providers (*n* = 7), opinion leaders (*n* = 12), and community members (57)	Thematic analysis	* unaccusable services (distance and cost) * Poor relationships among healthcare providers and women * Lack of privacy * insufficient follow up * The nurse is overworked and under-staffed	8
Mamba KC, 2017 (Mamba *et al*., 2017)	Malawi	To identify barriers that were inadvertently working against increasing PNC attendance in the first trimester	Structured interviews	NA	Thematic analysis	* unaffordable services	7
Maluka SO, 2020 (Maluka *et al.*, 2020)	Tanzania	To understand the factors leading to delay in seeking ANC services among pregnant women	Focus group discussions (FGDs) and semi-structured interviews	40 FGDs (with both male and female participants, 10-12 participants in each FGD), and 36 health workers	Thematic analysis	* Partner accompany policy * Rude language from health personnel * Shortage of healthcare providers	8
Manithip C, 2013 (Manithip *et al.*, 2013)	Laos	To explore the healthcare providers’ perceptions of the PNC services they provide	Semi-structured interviews	26 healthcare providers engaged in ANC services	Content analysis	* PNC providers are overloaded with work at the health centers * insufficient training regarding antenatal care among PNC providers * insufficient equipment * lack of motivation, feedback and support in terms of human resources	8
Titaley CR, 2010 (Titaley *et al.*, 2010)	Indonesia	To explore community members’ perspectives on antenatal and postnatal care services	Focus group discussions (FGDs) and in-depth interviews	295 community members	Content and thematic analysis	* perceived cost of health services * lesser quality of both health services and medications * Physical proximity to health services	8
Huaman Ayala LS, 2014 (Heaman *et al.*, 2014)	Peru	To investigate factors affecting pregnant women’s decision to seek or avoid antenatal care	Semi-structured interviews	24 women utilizing ANC and 10 women avoiding ANC	Content analysis	* long ANC wait time * inconvenient hours of operation * masculine gender of health workers	7
Baffour-Awuah A, 2015 (Baffour-Awuah *et al*., 2015)	Ghana	To explore the perceptions of midwives on focused antenatal care	Semi-structured interviews	40 Midwives	Content analysis	* lack of sufficient resources * workload * long waiting time * inadequate personal	8
Conrad P, 2012 (Conrad *et al.*, 2012)	Uganda	To understand how women experience the quality of ANC services	Semi structured interviews	30 pregnant women	Thematic analysis	* skipping some of the routine examinations by midwives * overburdened midwives * lack of adequate equipment, drugs, and reagents * not providing any pregnancy related education during ANC visits * providers arrive late or are absent without prior notice * Healthcare providers are not available at night or during weekends * pregnant women complained of rude midwives * healthcare providers do not report lab tests and do not explain the steps ahead in the care	8
Gross K, 2011 (Gross *et al.*, 2011)	Tanzania	To study the interplay between policy, context, and practice and its effect on PNC provision	Observations, informal conversations, in-depth interview	5 health workers	A mix of inductive and deductive category building	* Lack of needed drugs and supplied (e.g., for laboratory investigations) * Unavailable PNC guidelines * lack of effective documentation of pregnant women’s information * strict roles and routines and sanctioning the women for their non-compliant behavior	8
Graner S, 2010 (Graner *et al.*, 2010)	Vietnam	To study the perspectives of midwives and PNC providers on the content and quality of PNC	Four focus group discussions	21 midwives and 29 PNC providers	Latent content analysis	* inadequate facilities * inadequate human resources * inadequate professional re-training * Shortage of appropriate gloves and sterilizers * lonely work * long work hours * few possibilities for collegial support even during obstetrical emergencies	8
Larsen G, 2004 (Larsen *et al.*, 2004)	Papua New Guinea	To identify perceptions, barriers and strengths regarding the utilization of PNC	Semi-structured interviews and FGDs	20 pregnant or parous women and 4 PNC providers	NA	* geographical availability * long waiting times * negative attitudes of healthcare workers * occasional and unannounced closures of clinics * antenatal healthcare worker absents * lack of nutritional supplements or medications	7
Mahiti GR, 2015 (Mahiti *et al.*, 2015)	Tanzania	To explore women’s views about the PNC	Focus group discussions	105 women attending a health facility after child birth	content analysis	* lack of geographical access * shortage of human resources * multitasked nurses * lack of good PNC provider-utilizer relationship * Long waiting times * Instances of informal payments * drug shortages * dirty environment of healthcare facilities	6
Mathole T, 2005 (Mathole *et al*., 2005)	Zimbabwe	To investigate THE experiences of PNC providers, in caring for pregnant women	Individual interviews	18 nurses and midwives	---	* overworked healthcare providers * staff shortages * ambiguous job descriptions * drug shortage * poor quality of service * shortage of ambulances * cost of care	8
Myer L, 2003 (Meyer *et al.*, 2016)	South Africa	To document perceptions of PNC and to investigate factors shaping the utilization of PNC	Semi-structured interviews	29 pregnant women	Thematic analysis	* Lack of physical access to PNC clinics	7
Rahmani Z, 2013 (Rahmani & Brekke, 2013)	Afghanistan	To investigate how pregnant women and healthcare providers experience the existing PNC	Semi-structured interviews	12 women who were pregnant or had recently given birth and 15 healthcare workers	Giorgi’s phenomenological analysis	* Lack of professional ethical standards * Poor working conditions contribute to poor behavior * corruption in PNC clinics	8
Shabila NP, 2014 (Shabila *et al*., 2014)	Iraq	To explore the views and experiences of antenatal care in a sample of women	Q methodology	38 women	By-person factor analysis	* lack of convenient waiting amenities * unavailability of laboratory and ultrasound investigations * weak communication skills among PNC providers * receiving inadequate information during antenatal care visits * long waiting time * Pregnant women were usually seen by a nurse rather than a doctor * conflicting advice from different care providers	9
Uldbjerg CS, 2020 (Uldbjerg *et al.*, 2020)	Uganda	To identify perceived barriers to utilization of ANC services	In-depth interviews and focus group discussions	17 participants (13 pregnant women, 3 health workers, and 1 traditional birth attendant)	Inductive conventional content analysis	* Lack of resources at health centers * Poor attitude of health workers * Poor acceptance of cultural practices and beliefs at health centers * Compulsory HIV testing	9
Callaghan-Koru, 2016 (Callaghan-Koru *et al.*, 2016)	Tanzania	To explore providers’ communication about ANC visits and identify barriers to completing four visits	In-depth interviews (with providers) and exit interviews (with clients)	30 PNC providers and 203 PNC clients	Framework analysis	* Recommendation to bring male partners to ANC clinics for HIV testing * Out of pocket costs * Women turned away from services during first trimester * Poor provider communication about ANC visit schedule * Poor quality of care: long wait times, harsh treatment by providers, stock outs of drugs and tests * Long distances between homes and health facilities * Scheduling of specific dates for return ANC visits	
Chimatiro CS, 2018 (Chimatiro *et al.*, 2018)	Malawi	To explore barriers contributing to low utilization of PNC during the first trimester of pregnancy	In-depth interviews and Focus Group Discussions	10 PNC clients, 9 key informants, 3 health services professionals, and 126 women of child-bearing age (15–49 years)	Thematic analysis	* Long distances from home to the facility * Poor attitude of health workers * Long waiting time	9
Jacobs C, 2018 (Jacobs *et al*., 2018)	Zambia	To explain why one ANC visit with a skilled provider seemed more common than four ANC visits among women	Focus group discussions	38 Mothers, 28 community health volunteers, and 16 key informants	Thematic analysis	* Unavailable and Poor-quality services in the health posts * Inadequate supplies * Unavailable/Inadequate skilled healthcare providers * Long distances to the health facilities	8
Manda-Taylor, 2017 (Manda-Taylor *et al.*, 2017)	Malawi	To identify barriers to antenatal care uptake	Semi-structured interviews	12 pregnant mothers and 8 health workers	Thematic content analysis	* Insufficient data management * Sometimes women received ANC without any health education if they arrived early in the day * Limited counseling and testing room * lack of a consistent system to educate clients * women who attended late received no health education at all * lack of mid-career training for PNC providers	8
Nyathi L, 2017 (Nyathi *et al.*, 2017)	Zimbabwe	To investigate the accessibility factors influencing the use of PNC	Semi-structured interviews	15 mothers	Thematic analysis	* Long distance to the health facility * Poor healthcare workers’ attitude * not providing advice about pregnancy care * long waiting times	8
Nachinab, 2019 (Nachinab *et al.*, 2019)	Ghana	To explore the determinants of PNC uptake among women who failed to utilize PNC services	Face-to-face interview	15 mothers who did not attend ANC clinic	Inductive thematic analysis	* poorly equipped PNC clinics * negative attitude of PNC providers	8
Mgata S, 2019 (Mgata & Maluka, 2019)	Tanzania	To understand factors that lead to the delay in seeking ANC services among pregnant	In-depth interviews	20 pregnant women and 5 healthcare workers	Thematic analysis	* Distance to the health facility * Required escort of a spouse during the first ANC visit * shortage of health providers	8
Meyer, 2016 (Myer & Harrison, 2003)	Georgia	To identify the PNC access barriers experienced by women	Semi-structured, in-depth interviews	24 mothers and 4 key informant	Applied thematic analysis	* difficulties in locating providers with their preferred criteria * insufficient health insurance coverage * Lack of consistency and continuity in PNC care * poor communication among healthcare providers and clients	9

### Overview of included studies

Of the 32 included studies, 25 (78%) were published either in or after 2013. The studies took place in 21 countries across four continents. Of the included studies, 59% discussed countries or regions in Africa, with Tanzania and Malawi being the most common of these; 25% discussed Asian countries or regions and only one study (3.1%); and discussed barriers in the South America and one in Papua New Guinea from Oceania (3.1%).

Numbers of participants varied from five to 295, with most between 20 and 80 participants. The total population sample included 1677 participants including 629 pregnant women, 122 mothers, 240 healthcare providers, 54 key informed, 164 women of child bearing age, 380 community members, and 88 participants from other groups (such as key informants or male partners).

### Quality of studies

The overall quality assessment of the studies was conducted by rating CASP items (Table 2). All of them had a clear statement of the research objectives and appropriate qualitative methodology (the first two essential items of CASP); thus, no study was excluded due to quality issue.

### Overview of health system barriers identified

**Figure 2.**
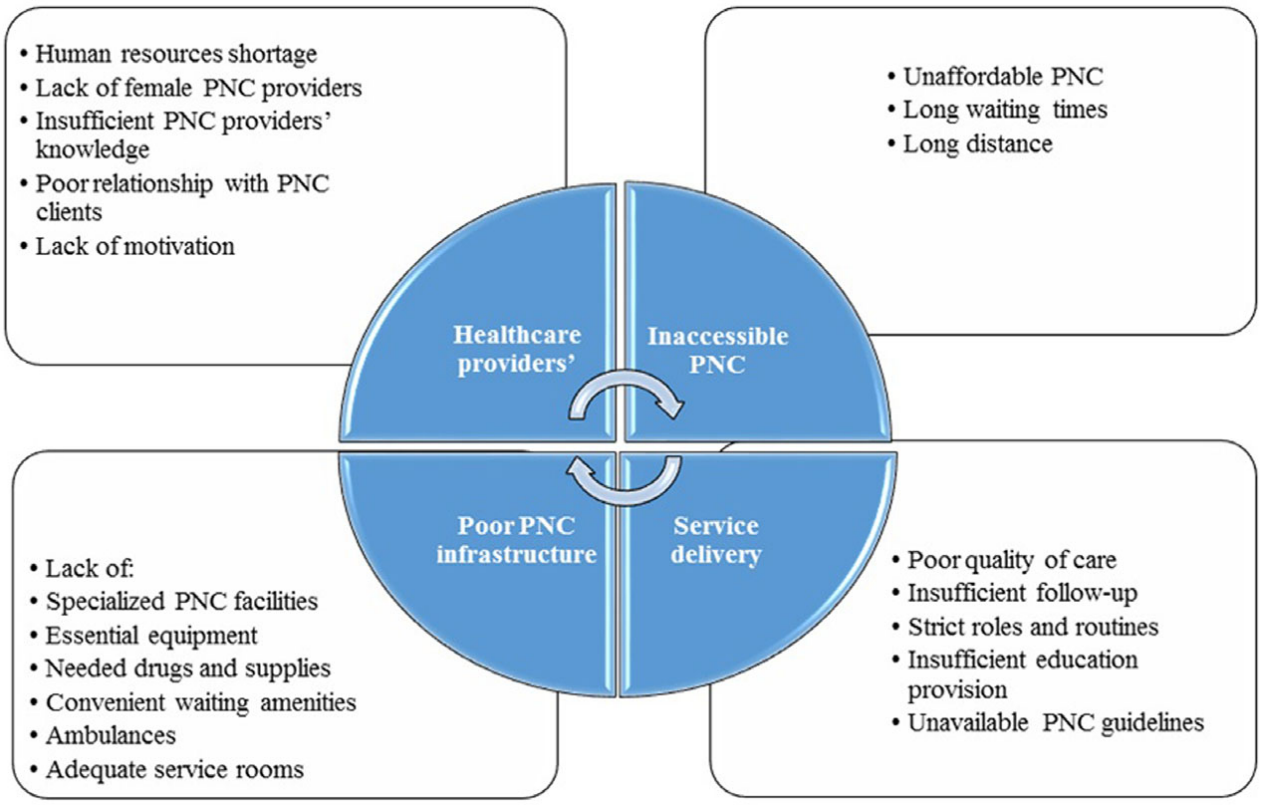
Health system-related barriers to prenatal care management

We categorized the review findings into four main themes: healthcare provider-related issues, service delivery issues, inaccessible PNC, and poor PNC infrastructure. There are one to five subthemes under each theme that are presented in Figure 2 and Table 3.

**Table 3.** Thematic analysis

Main themes	Subthemes	References
1. Healthcare providers’	Human resources shortage	(Larsen *et al.*, 2004; Conrad *et al.*, 2012; Rahmani & Brekke, 2013; Andrew *et al.*, 2014; Mahiti *et al.*, 2015; Callaghan-Koru *et al.*, 2016; Meyer *et al.*, 2016; Nyathi *et al.*, 2017; Chimatiro *et al.*, 2018; Alanazy *et al.,* 2019; Nachinab *et al.*, 2019; Maluka *et al.*, 2020; Uldbjerg *et al.*, 2020; Udenigwe *et al.*, 2021)
	Lack of female PNC providers	(Ayala *et al.*, 2013; Akter *et al.*, 2018)
	Insufficient PNC providers’ knowledge	(Graner *et al.*, 2010; Manithip *et al.*, 2013; Manda-Taylor *et al.*, 2017)
	Poor relationship with PNC clients	(Larsen *et al.*, 2004; Conrad *et al.*, 2012; Rahmani & Brekke, 2013; Andrew *et al.*, 2014; Mahiti *et al.*, 2015; Callaghan-Koru *et al.*, 2016; Meyer *et al.*, 2016; Nyathi *et al.*, 2017; Chimatiro *et al.*, 2018; Alanazy *et al.*, 2019; Nachinab *et al.*, 2019; Maluka *et al.*, 2020; Uldbjerg *et al.*, 2020; Mourtada *et al*., 2021; Tsegaye *et al.*, 2021; Udenigwe *et al*., 2021)
	Lack of motivation	(Larsen *et al.*, 2004; Conrad *et al.*, 2012; Manithip *et al.*, 2013)
2. Service delivery	Poor quality of care	(Mathole *et al.*, 2005; Titaley *et al.*, 2010; Conrad *et al.*, 2012; Alanazy *et al*., 2019)
	Insufficient follow-up	(Myer & Harrison, 2003; Andrew *et al.*, 2014)
	Strict roles and routines	(Gross *et al.*, 2011; Callaghan-Koru *et al.*, 2016; Manda-Taylor *et al.*, 2017; Mgata & Maluka, 2019; Maluka *et al.*, 2020; Uldbjerg *et al.*, 2020; Udenigwe *et al.*, 2021; Hajian *et al*., 2022)
	Insufficient education provision	(Conrad *et al.*, 2012; Shabila *et al.*, 2014; Manda-Taylor *et al*., 2017; Nyathi *et al.*, 2017)
	Unavailable PNC guidelines	(Larsen *et al.*, 2004; Titaley *et al.*, 2010; Andrew *et al.*, 2014; Mahiti *et al.*, 2015; Callaghan-Koru *et al.*, 2016; Meyer *et al.*, 2016; Nyathi *et al.*, 2017; Chimatiro *et al.*, 2018; Jacobs *et al.*, 2018; Mgata & Maluka, 2019)
3. Inaccessible PNC	Unaffordable PNC	(Mathole *et al.*, 2005; Titaley *et al.*, 2010; Rahmani & Brekke, 2013; Andrew *et al.*, 2014; Mahiti *et al.*, 2015; Callaghan-Koru *et al.*, 2016; Meyer *et al.*, 2016; Mamba *et al.*, 2017; Akter *et al.*, 2018; Dadras *et al.*, 2020; Tsegaye *et al.*, 2021; Udenigwe *et al.*, 2021)
	Long waiting times	(Larsen *et al.*, 2004; Ayala *et al.,* 2013; Shabila *et al.*, 2014; Baffour-Awuah *et al.*, 2015; Mahiti *et al.*, 2015; Callaghan-Koru *et al.*, 2016; Nyathi *et al.*, 2017; Chimatiro *et al.*, 2018; Alanazy *et al*., 2019)
	Long distance	(Larsen *et al.*, 2004; Titaley *et al.*, 2010; Andrew *et al.*, 2014; Mahiti *et al.*, 2015; Callaghan-Koru *et al.*, 2016; Meyer *et al.*, 2016; Nyathi *et al.*, 2017; Chimatiro *et al.*, 2018; Jacobs *et al.*, 2018; Mgata & Maluka, 2019)
4. Poor PNC infrastructure	Lack of: • Specialized PNC facilities • Essential equipment • Needed drugs and supplies • Convenient waiting amenities • Ambulances • Adequate service rooms	(Larsen *et al.*, 2004; Mathole *et al.*, 2005; Graner *et al.*, 2010; Gross *et al.*, 2011; Conrad *et al.*, 2012; Manithip *et al.*, 2013; Shabila *et al.*, 2014; Baffour-Awuah *et al*., 2015; Mahiti *et al.*, 2015; Callaghan-Koru *et al.*, 2016; Manda-Taylor *et al*., 2017; Jacobs *et al.*, 2018; Alanazy *et al.*, 2019; Nachinab *et al.*, 2019; Uldbjerg *et al.*, 2020; Tsegaye *et al.*, 2021)

### Theme 1: healthcare provider-related issues

Concerns about the negative impact of healthcare providers’ issues on the PNC emerged as a prominent theme with five subthemes: (1) human resource shortage; (2) lack of female PNC providers; (3) insufficient PNC providers’ knowledge; (4) poor relationship with PNC clients; and (5) lack of motivation.

#### Human resource shortage

Participants in many of included studies expressed concerns over insufficient human resources (Mathole *et al.*, 2005; Graner *et al.*, 2010; Andrew *et al.*, 2014; *Baffour-Awuah et al*., 2015; Mahiti *et al.*, 2015; Mgata & Maluka, 2019; Maluka *et al.*, 2020; Udenigwe *et al.*, 2021). They believed that this PNC shortage makes PNC providers overloaded with work (Mathole *et al.*, 2005; Conrad *et al.*, 2012; Manithip *et al.*, 2013; Andrew *et al.*, 2014; Baffour-Awuah *et al*., 2015; Mahiti *et al.*, 2015; Alanazy *et al*., 2019).

#### Lack of female PNC provider

Finding of this review indicates that lack of female PNC provider is a significant barrier to PNC in LMICs. Some article indicted that one of the important reasons for women to not seek PNC was feeling embarrassed, discomfort, and mistrust about having a male health worker (Ayala *et al*., 2013; Akter *et al.*, 2018).

#### Insufficient PNC providers’ knowledge

Stakeholders believed that availability of skilled and well-trained healthcare providers is an important requisite for provision of quality PNC. However, some of included studies reported insufficient trainings regarding PNC among healthcare providers (Manithip *et al.*, 2013). According to the participants’ points of view, healthcare providers do not receive sufficient professional retraining (Graner *et al.*, 2010; Manda-Taylor *et al.*, 2017).

#### Poor relationship with PNC clients

Stakeholders perceived lack of a good relationship between healthcare providers and PNC clients as a key barrier to PNC (Larsen *et al.*, 2004; Conrad *et al.*, 2012; Rahmani & Brekke, 2013; Andrew *et al.*, 2014; Mahiti *et al.*, 2015; Callaghan-Koru *et al.*, 2016; Meyer *et al.*, 2016; Alanazy *et al*., 2019; Maluka *et al.*, 2020; Uldbjerg *et al.*, 2020; Mourtada *et al*., 2021; Tsegaye *et al*., 2021; Udenigwe *et al*., 2021). Some of them reported that PNC providers have negative attitudes toward PNC clients (Larsen *et al.*, 2004; Nyathi *et al.*, 2017; Chimatiro *et al.*, 2018; Nachinab *et al.*, 2019; Uldbjerg *et al.*, 2020) and they refuse to consider them seriously (Alanazy *et al*., 2019). Participants in some of included studies even stated that they were treated rudely by PNC providers (Conrad *et al.*, 2012; Maluka *et al.*, 2020).

#### Lack of motivation

There was also some evidence that there is lack of motivation and satisfaction among healthcare workers (Manithip *et al.*, 2013). Participants believed that because of this problem, providers arrive late or are absent without any prior notice (Larsen *et al.*, 2004; Conrad *et al.*, 2012) and clients experience occasional and unannounced closures of clinics (Larsen *et al.*, 2004).

### Theme 2: service delivery issues

Stakeholders participated in the included studies constantly described service delivery issues as important barriers to PNC. There were five subthemes related to this theme:

#### Poor quality of care

According to some participants’ point of view in several included studies, barriers regarding the poor quality of care hinder PNC provision/utilization (Mathole *et al*., 2005; Titaley *et al.*, 2010; Alanazy *et al*., 2019). They believed that sometimes essential PNC procedures such as routine examinations were skipped during the PNC visits (Conrad *et al.*, 2012). They also mentioned that the PNC process is not transparent and healthcare providers do not explain the steps ahead in the care (Conrad *et al.*, 2012). Some participants even complained that PNC clients do not receive lab tests results (Conrad *et al.*, 2012).

#### Insufficient follow-up

One of the perceived barriers regarding PNC management in LMICs was lack of sufficient follow-up to ensure continuity of care (Andrew *et al.*, 2014). Participants believed that this factor can lead to discontinuity in PNC (Myer & Harrison, 2003).

#### Strict roles and routines

One of the commonly mentioned barriers to PNC was strict roles and routines in PNC clinics such as partner accompany policy (Gross *et al.*, 2011; Callaghan-Koru *et al.*, 2016; Mgata & Maluka, 2019; Maluka *et al.*, 2020) or compulsory HIV testing (Uldbjerg *et al.*, 2020) and sanctioning the PNC clients because of their noncompliant behavior. Scheduling of specific dates for return PNC visits was mentioned as another strict role hindering PNC utilization (Callaghan-Koru *et al.*, 2016; Hajian *et al.*, 2022; Udenigwe *et al*., 2021). Some of studies even reported that clients who attend earlier or later than predefined times will never receive PNC education (Manda-Taylor *et al*., 2017).

#### Insufficient education provision

The participants widely reported that PNC clients have not been provided with the necessary knowledge and training (Conrad *et al.*, 2012; Shabila *et al*., 2014; Nyathi *et al.*, 2017). Some of respondents in included studies highlighted that there is not a consistent system to do this important component of PNC management (Manda-Taylor *et al.*, 2017).

#### Unavailable PNC guidelines

Lack of evidence-based PNC guidelines was highlighted by participants as a major barrier regarding PNC (Larsen *et al.*, 2004; Titaley *et al.*, 2010; Andrew *et al.*, 2014; Mahiti *et al.*, 2015; Callaghan-Koru *et al.*, 2016; Meyer *et al.*, 2016; Nyathi *et al.*, 2017; Chimatiro *et al.*, 2018; Jacobs *et al*., 2018; Mgata & Maluka, 2019).

### Theme 3: inaccessible PNC

The theme of inaccessible PNC emerged to organize barriers related to different aspects of PNC accessibility. This theme emerged from three categories including (1) long distance; (2) unaffordable PNC; and (3) long waiting times.

#### Unaffordable PNC

Many of participants believed that pregnant women cannot afford the cost of PNC. They reported high cost of care, laboratory tests, and medications (Mathole *et al*., 2005; Titaley *et al.*, 2010; Andrew *et al.*, 2014; Mamba *et al*., 2017; Akter *et al.*, 2018) most of which should be paid out of pocket as a result of insufficient health insurance coverage (Callaghan-Koru *et al.*, 2016; Meyer *et al.*, 2016). We found that corruptions in PNC clinics and instance of informal payment being demanded from clients pose additional barrier in terms of financial accessibility (Rahmani & Brekke, 2013; Mahiti *et al.*, 2015). In addition, financial constraints were highlighted by some participants as a barrier regarding PNC (Dadras *et al.*, 2020; Tsegaye *et al.*, 2021; Udenigwe *et al.*, 2021)

#### Long waiting times

Waiting time was another important accessibility area in which frustration was expressed. The participants believed that long waiting times would be the factor which would discourage pregnant women from seeking PNC services (Larsen *et al.*, 2004; Ayala *et al*., 2013; Shabila *et al*., 2014; Baffour-Awuah *et al*., 2015; Mahiti *et al.*, 2015; Callaghan-Koru *et al.*, 2016; Nyathi *et al.*, 2017; Chimatiro *et al.*, 2018; Alanazy *et al*., 2019).

#### Long distances

According to participants’ perspectives, geographical access to PNC appears inadequate. They mentioned that PNC seekers’ access to care is restricted by long distance (Larsen *et al.*, 2004; Titaley *et al.*, 2010; Andrew *et al.*, 2014; Mahiti *et al.*, 2015; Callaghan-Koru *et al.*, 2016; Meyer *et al.*, 2016; Nyathi *et al.*, 2017; Chimatiro *et al.*, 2018; Jacobs *et al*., 2018; Mgata & Maluka, 2019).

### Theme 4: poor PNC infrastructure

We found that many of participants complained that poor PNC clinic facilities hindered PNC provision or utilization (Larsen *et al.*, 2004; Mathole *et al*., 2005; Graner *et al.*, 2010; Gross *et al.*, 2011; Conrad *et al.*, 2012; Manithip *et al.*, 2013; Shabila *et al.*, 2014; Baffour-Awuah *et al.*, 2015; Mahiti *et al.*, 2015; Callaghan-Koru *et al.*, 2016; Manda-Taylor *et al*., 2017; Jacobs *et al*., 2018; Alanazy *et al.*, 2019; Nachinab *et al.*, 2019; Uldbjerg *et al.*, 2020). They mentioned long list of infrastructure-related barriers including lack of specialized PNC facilities (Alanazy *et al*., 2019), lack of sufficient resources (Baffour-Awuah *et al.*, 2015), such as essential equipment such as appropriate gloves and sterilizers (Conrad *et al.*, 2012; Manithip *et al.*, 2013; Shabila *et al*., 2014; Nachinab *et al.*, 2019), needed drugs and supplies (Larsen *et al.*, 2004; Mathole *et al.*, 2005; Gross *et al.*, 2011; Conrad *et al.*, 2012; Callaghan-Koru *et al.*, 2016; Jacobs *et al*., 2018; Tsegaye *et al.*, 2021), ambulances (Mathole *et al.*, 2005), convenient waiting amenities (Shabila *et al.*, 2014), adequate service rooms such as counseling and testing room (Manda-Taylor *et al.*, 2017), and clean PNC clinics’ environment (Mahiti *et al.*, 2015).

## Discussion

PNC is an essential component of improving maternal and infant health during pregnancy and birth, by treating and monitoring potential complications. This review set out to summarize the qualitative literature concerning the healthcare system-related barriers in PNC management in LMICs. Included studies came from a variety of countries and help understand the range of different potential difficulties in PNC management from several continents. Findings of this systematic review suggest that PNC in LMICs can be challenged by a number of barriers at different levels of healthcare systems, including human resources aspects, service delivery issues, PNC accessibility, and PNC infrastructures.

In addition to a wide range of countries with low- and middle-income settings, the included studies encompassed a wide range data from different types of PNC stakeholders such as healthcare providers, pregnant women, male partners, and community members. This indicates that PNC stakeholders, in any role, are aware that PNC is provided in a context lead by the healthcare system.

It is notable that the majority of barriers identified within the evidence emerged within the human resources and service delivery themes. This stakeholder perception is supported by other systematic reviews investigating LMICs barriers in other maternal health contexts such as midwifery care (Filby *et al*., 2016). In addition, many of WHO’s healthcare system-related recommendations on PNC improvement could be mapped directly to some of the findings identified in this systematic review. These were mainly to do with continuity of care, communication, and PNC contact schedule (World Health Organization, 2021). One of the main results that was not considered seriously in this recommendation was attitudes and behaviors of healthcare staff. This issue is also ignored in some other effectiveness studies in the area of antenatal care design and provision (Finlayson & Downe, 2013; Downe *et al.*, 2016). This seems to be an important omission.

Many of the emerged barriers in this review of qualitative studies also match those observed in earlier quantitative studies. For example, one of them highlighted insufficient geographical accessibility (Kuupiel *et al.*, 2020). The findings of previous quantitative studies also suggest a need to cultivate quality of PNC care (Sommer Albert *et al*., 2020), train PNC providers in communication skills (Sommer Albert *et al*., 2020), and expand technical capacity by continuing education and supportive supervision to train PNC providers to follow standard protocols for provision of quality ANC services (Sommer Albert *et al.*, 2020). We recommend that the results of this review should be considered when implementing PNC strategies in LMICs and other low resource settings.

### Strengths and limitations of this review

This review provides a comprehensive approach to qualitative studies of healthcare system-related barriers to PNC in LMICs. Exploring pregnant women, PNC providers, and general population accounts also provided a rounded understanding of PNC barriers from multiple perspectives.

There are several important limitations to note when interpreting the results of this review. One limitation is that it we only included articles published in English, which may suggest that the potentially relevant studies from cultural contexts where English is not the norm may be missed. In addition, limited time and resources prevented a more thorough and comprehensive search of the gray literature, a body of evidence that may have had more to offer PNC clients’ experiences and perspectives.

### Gaps in the evidence base

Despite all of the works that has been conducted in the area of PNC barriers, the current review noted a significant gap in the evidence base related to PNC and healthcare systems. This important gap is the perspectives of women who are underrepresented in the data: pregnant women who did not make it to PNC. Because of health system-centric nature of the majority of related literature, there is much more information about pregnant women who stayed in care than about those who never attend PNC.

## Conclusion

This review contributes to the current debate on the knowledge of key barriers to PNC in LMICs contexts. Findings of this systematic review suggest that PNC in LMICs can be challenged by a number of barriers at different levels of healthcare systems, including human resources aspects, service delivery issues, PNC accessibility, and PNC infrastructures. Healthcare policymakers in LMICs, when planning and managing the PNC, should consider the lessons learnt from previous reports as synthesized in this review and should carefully develop strategies to prevent and mitigate common barriers to successful PNC.

## References

[ref1] Adane AA , Ayele TA , Ararsa LG , Bitew BD and Zeleke BM (2014) Adverse birth outcomes among deliveries at Gondar University Hospital, Northwest Ethiopia. BMC Pregnancy and Childbirth 14, 90. 24576205 10.1186/1471-2393-14-90 PMC3996071

[ref2] Akter MK , Yimyam S , Chareonsanti J and Tiansawad S (2018) The challenges of prenatal care for Bangladeshi women: a qualitative study. International Nursing Review 65, 534–541. 30176049 10.1111/inr.12466

[ref3] Alanazy W , Rance J and Brown A (2019) Exploring maternal and health professional beliefs about the factors that affect whether women in Saudi Arabia attend antenatal care clinic appointments. Midwifery 76, 36–44. 31154158 10.1016/j.midw.2019.05.012

[ref4] Andrew EV , Pell C , Angwin A , Auwun A , Daniels J , Mueller I , Phuanukoonnon S and Pool R (2014) Factors affecting attendance at and timing of formal antenatal care: results from a qualitative study in Madang, Papua New Guinea. PLoS ONE 9, e93025. 24842484 10.1371/journal.pone.0093025 PMC4026245

[ref5] Ayala LSH , Blumenthal PD and Sarnquist CC (2013) Factors influencing women’s decision to seek antenatal care in the Andes of Peru. Maternal and Child Health Journal 17, 1112–1118. 22956365 10.1007/s10995-012-1113-9

[ref6] Azmat SK , Marleen T and Moazzam A (2021) Accessibility and uptake of modern contraceptive methods in Pakistan-a critical view on what works? The Journal of the Pakistan Medical Association 71, S20–S32. 34793425

[ref7] Baffour-Awuah A , Mwini-Nyaledzigbor PP and Richter S (2015) Enhancing focused antenatal care in Ghana: an exploration into perceptions of practicing midwives. International Journal of Africa Nursing Sciences 2, 59–64.

[ref8] Callaghan-Koru JA , McMahon SA , Chebet JJ , Kilewo C , Frumence G , Gupta S , Stevenson R , Lipingu C , Baqui AH and Winch PJ (2016) A qualitative exploration of health workers’ and clients’ perceptions of barriers to completing four antenatal care visits in Morogoro Region, Tanzania. Health Policy and Planning 31, 1039–1049. 27117481 10.1093/heapol/czw034

[ref9] CASP UK (2018) CASP Checklist: 10 questions to help you make sense of a Qualitative research. Retrieved 1 October 2022 from https://casp-uk.net/casp-tools-checklists.

[ref10] Chimatiro CS , Hajison P , Chipeta E and Muula AS (2018) Understanding barriers preventing pregnant women from starting antenatal clinic in the first trimester of pregnancy in Ntcheu District-Malawi. Reproductive Health 15, 158. 30241542 10.1186/s12978-018-0605-5 PMC6151039

[ref11] Chowdhury MAK , Karim F , Hasan MM , Ali NB , Khan ANS , Siraj MS , Ahasan SM and Hoque DME (2022) Bottleneck analysis of maternal and newborn health services in hard-to-reach areas of Bangladesh using “TANAHASHI’framework”: an explanatory mixed-method study. PLoS ONE 17, e0268029. 35551544 10.1371/journal.pone.0268029 PMC9098042

[ref12] Cisse S , Rossier C and Sauvain-Dugerdil C (2022) Women’s personal networks and recourse to prenatal care in Bamako. Journal of Demographic Economics 88, 195–216.

[ref13] Conrad P , De Allegri M , Moses A , Larsson EC , Neuhann F , Müller O and Sarker M (2012) Antenatal care services in rural Uganda: missed opportunities for good-quality care. Qualitative Health Research 22, 619–629. 22232296 10.1177/1049732311431897

[ref14] Dadras O , Taghizade Z , Dadras F , Alizade L , Seyedalinaghi S , Ono-Kihara M , Kihara M and Nakayama T (2020) “It is good, but I can’t afford it…” potential barriers to adequate prenatal care among Afghan women in Iran: a qualitative study in South Tehran. BMC Pregnancy and Childbirth 20, 1–10. 10.1186/s12884-020-02969-x PMC7201652 32375696

[ref15] Dattilo AM , Carvalho RS , Feferbaum R , Forsyth S and Zhao A (2020) ‘Hidden realities of infant feeding: systematic review of qualitative findings from parents. Behavioral Sciences 10, 83. 32349324 10.3390/bs10050083 PMC7287829

[ref16] Downe S , Finlayson K , Tunçalp Ó and Metin Gülmezoglu A (2016) What matters to women: a systematic scoping review to identify the processes and outcomes of antenatal care provision that are important to healthy pregnant women. BJOG: An International Journal of Obstetrics and Gynaecology 123, 529–539. 26701735 10.1111/1471-0528.13819

[ref17] Fagbamigbe AF and Idemudia ES (2015) Assessment of quality of antenatal care services in Nigeria: evidence from a population-based survey. Reproductive Health 12, 88. 26382228 10.1186/s12978-015-0081-0 PMC4574449

[ref18] Filby A , McConville F and Portela A (2016) What prevents quality midwifery care? A systematic mapping of barriers in low and middle income countries from the provider perspective. PLoS ONE 11, e0153391. 27135248 10.1371/journal.pone.0153391 PMC4852911

[ref19] Finlayson K and Downe S (2013) Why do women not use antenatal services in low-and middle-income countries? A meta-synthesis of qualitative studies. PLoS Medicine 10, e1001373. 23349622 10.1371/journal.pmed.1001373 PMC3551970

[ref20] Graner S , Mogren I , Duong LQ , Krantz G and Klingberg-Allvin M (2010) Maternal health care professionals’ perspectives on the provision and use of antenatal and delivery care: a qualitative descriptive study in rural Vietnam. BMC Public Health 10, 608. 20946681 10.1186/1471-2458-10-608 PMC3091560

[ref21] Gross K , Schellenberg JA , Kessy F , Pfeiffer C and Obrist B (2011) Antenatal care in practice: an exploratory study in antenatal care clinics in the Kilombero Valley, south-eastern Tanzania. BMC Pregnancy Childbirth 11, 36. 21599900 10.1186/1471-2393-11-36 PMC3123249

[ref22] Hadden KB (2012) Health literacy and pregnancy: validation of a new measure and relationships of health literacy to pregnancy risk factors. Little Rock, Arkansas: University of Arkansas for Medical Sciences.

[ref23] Hajian S , Mehran N , Simbar M and Alavi Majd H (2022) The barriers and facilitators of Iranian men’s involvement in perinatal care: a qualitative study. Reproductive Health 19, 1–9. 35189902 10.1186/s12978-022-01350-9 PMC8862297

[ref24] Heaman MI , Green CG , Newburn-Cook CV , Elliott LJ and Helewa ME (2007) Social inequalities in use of prenatal care in Manitoba. Journal of Obstetrics and Gynaecology Canada 29, 806–816. 17915064 10.1016/s1701-2163(16)32637-8

[ref25] Heaman MI , Moffatt M , Elliott L , Sword W , Helewa ME , Morris H , Gregory P , Tjaden L and Cook C (2014) Barriers, motivators and facilitators related to prenatal care utilization among inner-city women in Winnipeg, Canada: a case-control study. BMC Pregnancy and Childbirth 14, 227. 25023478 10.1186/1471-2393-14-227 PMC4223395

[ref26] Jacobs C , Michelo C and Moshabela M (2018) Why do rural women in the most remote and poorest areas of Zambia predominantly attend only one antenatal care visit with a skilled provider? A qualitative inquiry. BMC Health Services Research 18, 409. 29871624 10.1186/s12913-018-3212-9 PMC5989442

[ref27] Joseph J , Brodribb W and Liamputtong P (2019) “Fitting-in Australia” as nurturers: meta-synthesis on infant feeding experiences among immigrant women. Women and Birth 32, 533–542. 30580993 10.1016/j.wombi.2018.12.002

[ref28] Krukowski RA , Jacobson LT , John J , Kinser P , Campbell K , Ledoux T , Gavin KL , Chiu C-Y , Wang J and Kruper A (2022) Correlates of early prenatal care access among us women: data from the pregnancy risk assessment monitoring system (PRAMS). Maternal and Child Health Journal 26, 328–341. 34606031 10.1007/s10995-021-03232-1 PMC8488070

[ref29] Kuupiel D , Adu KM , Bawontuo V , Adogboba DA , Drain PK , Moshabela M and Mashamba-Thompson TP (2020) Geographical accessibility to glucose-6-phosphate dioxygenase deficiency point-of-care testing for antenatal care in Ghana. Diagnostics 10, 229. 32316233 10.3390/diagnostics10040229 PMC7235997

[ref30] Larsen G , Lupiwa S , Kave H , Gillieatt S and Alpers M (2004) Antenatal care in Goroka: issues and perceptions. Papua New Guinea Medical Journal 47, 202–214. 16862944

[ref31] Lassi ZS , Middleton P , Bhutta ZA and Crowther C (2019) Health care seeking for maternal and newborn illnesses in low- and middle-income countries: a systematic review of observational and qualitative studies. F1000Research 8, 200. 31069067 10.12688/f1000research.17828.1 PMC6480947

[ref32] Mahiti GR , Mkoka DA , Kiwara AD , Mbekenga CK , Hurtig A-K and Goicolea I (2015) Women’s perceptions of antenatal, delivery, and postpartum services in rural Tanzania. Global Health Action 8, 28567. 26498576 10.3402/gha.v8.28567 PMC4617868

[ref33] Maluka SO , Joseph C , Fitzgerald S , Salim R and Kamuzora P (2020) Why do pregnant women in Iringa region in Tanzania start antenatal care late? A qualitative analysis. BMC Pregnancy Childbirth 20, 126. 32093645 10.1186/s12884-020-2823-4 PMC7041254

[ref34] Mamba KC , Muula AS and Stones W (2017) Facility-imposed barriers to early utilization of focused antenatal care services in Mangochi District, Malawi – a mixed methods assessment. BMC Pregnancy Childbirth 17, 444. 29284439 10.1186/s12884-017-1631-y PMC5747179

[ref35] Manda-Taylor L , Sealy DA and Roberts J (2017) Factors associated with delayed antenatal care attendance in Malawi: results from a qualitative study. Medical Journal of Zambia 44, 17–25.

[ref36] Manithip C , Edin K , Sihavong A , Wahlström R and Wessel H (2013) Poor quality of antenatal care services—is lack of competence and support the reason? An observational and interview study in rural areas of Lao PDR. Midwifery 29, 195–202. 22776568 10.1016/j.midw.2011.12.010

[ref37] Mathole T , Lindmark G and Ahlberg BM (2005) Dilemmas and paradoxes in providing and changing antenatal care: a study of nurses and midwives in rural Zimbabwe. Health Policy and Planning 20, 385–393. 16183736 10.1093/heapol/czi046

[ref38] McMaster University (2016) Search *filters for* MEDLINE in *ovid syntax and the pubmed translation*. Retrieved 5 October 2022 from https://hiru.mcmaster.ca/hiru/hiru_hedges_medline_strategies.aspx.

[ref39] Meyer E , Hennink M , Rochat R , Julian Z , Pinto M , Zertuche AD , Spelke B , Dott A and Cota P (2016) Working towards safe motherhood: delays and barriers to prenatal care for women in rural and peri-urban areas of Georgia. Maternal and Child Health Journal 20, 1358–1365. 27053128 10.1007/s10995-016-1997-x

[ref40] Mgata S and Maluka SO (2019) Factors for late initiation of antenatal care in Dar es Salaam, Tanzania: a qualitative study. BMC Pregnancy Childbirth 19, 415. 31718586 10.1186/s12884-019-2576-0 PMC6849280

[ref41] Mourtada R , Bashour H and Houben F (2021) A qualitative study exploring barriers to adequate uptake of antenatal care in pre-conflict Syria: low cost interventions are needed to address disparities in antenatal care. Contraception and Reproductive Medicine 6, 1–12. 34059151 10.1186/s40834-021-00156-7 PMC8167987

[ref42] Myer L and Harrison A (2003) Why do women seek antenatal care late? Perspectives from rural South Africa. Journal of Midwifery & Womens Health 48, 268–272. 10.1016/s1526-9523(02)00421-x 12867911

[ref43] Nachinab GT-e , Adjei CA , Ziba FA , Asamoah R and Attafuah PA (2019) Exploring the determinants of antenatal care services uptake: a qualitative study among women in a rural community in Northern Ghana. Journal of Pregnancy 2019, 3532749. 31929907 10.1155/2019/3532749 PMC6935810

[ref44] Nyathi L , Tugli AK , Tshitangano TG and Mpofu M (2017) Investigating the accessibility factors that influence antenatal care services utilisation in Mangwe district, Zimbabwe. African Journal of Primary Health Care & Family Medicine 9, 1–5. 10.4102/phcfm.v9i1.1337 PMC5506496 28697619

[ref45] Page MJ , McKenzie JE , Bossuyt PM , Boutron I , Hoffmann TC , Mulrow CD , Shamseer L , Tetzlaff JM , Akl EA and Brennan SE (2021) The PRISMA 2020 statement: an updated guideline for reporting systematic reviews. International Journal of Surgery 1, 105906. 10.1016/j.ijsu.2021.105906 33789826

[ref46] Panda B and Thakur HP (2016) Decentralization and health system performance–a focused review of dimensions, difficulties, and derivatives in India. BMC Health Services Research 16, 1–14. 28185593 10.1186/s12913-016-1784-9 PMC5103245

[ref47] Racine N , Byles H , Killam T , Ereyi-Osas W and Madigan S (2022) Asking about childhood adversity in the prenatal care setting: cross-sectional associations with maternal health and mental health outcomes. Maternal and Child Health Journal 26, 994–1004. 34837600 10.1007/s10995-021-03301-5

[ref48] Rahmani Z and Brekke M (2013) Antenatal and obstetric care in Afghanistan – a qualitative study among health care receivers and health care providers. BMC Health Services Research 13, 166. 23642217 10.1186/1472-6963-13-166 PMC3654902

[ref49] Shabila NP , Ahmed HM and Yasin MY (2014) Women’s views and experiences of antenatal care in Iraq: a Q methodology study. BMC Pregnancy Childbirth 14, 43. 24450437 10.1186/1471-2393-14-43 PMC3902000

[ref50] Sommer Albert J , Younas A and Victor G (2020) Quality of antenatal care services in a developing country: a cross-sectional survey. Creative Nursing 26, e25–e34. 32024745 10.1891/1078-4535.26.1.e25

[ref51] Thomas J and Harden A (2008) Methods for the thematic synthesis of qualitative research in systematic reviews. BMC Medical Research Methodology 8, 45. 18616818 10.1186/1471-2288-8-45 PMC2478656

[ref52] Titaley CR , Hunter CL , Heywood P and Dibley MJ (2010) Why don’t some women attend antenatal and postnatal care services? A qualitative study of community members’ perspectives in Garut, Sukabumi and Ciamis districts of West Java Province, Indonesia. BMC Pregnancy Childbirth 10, 61. 20937146 10.1186/1471-2393-10-61 PMC2964562

[ref53] Tsegaye ZT , Abawollo HS , Desta BF , Mamo TT , Heyi AF , Mesele MG and Lose AD (2021) Contributing barriers to loss to follow up from antenatal care services in villages around Addis Ababa: a qualitative study. BMC Women’s Health 21, 1–9. 33827513 10.1186/s12905-021-01290-9 PMC8028793

[ref54] Udenigwe O , Okonofua FE , Ntoimo LF , Imongan W , Igboin B and Yaya S (2021) Perspectives of policymakers and health providers on barriers and facilitators to skilled pregnancy care: findings from a qualitative study in rural Nigeria. BMC Pregnancy and Childbirth 21, 1–14. 33407238 10.1186/s12884-020-03493-8 PMC7789224

[ref55] Uldbjerg CS , Schramm S , Kaducu FO , Ovuga E and Sodemann M (2020) Perceived barriers to utilization of antenatal care services in northern Uganda: a qualitative study. Sexual & Reproductive Healthcare 23, 100464. 31710878 10.1016/j.srhc.2019.100464

[ref56] United Nations (2008) The Millennium *development goals report* 2008. Retrieved 5 October 2022 from https://www.un-kampagne.de/fileadmin/downloads/news3/MDG_Report_2008.pdf.

[ref57] United Nations (2021) The sustainable development goals report 2021. New York: UN.

[ref58] World Health Organization (2019) Maternal mortality: evidence brief. Geneva: WHO.

[ref59] World Health Organization (2021) WHO antenatal care recommendations for a positive pregnancy experience: nutritional interventions update: zinc supplements during pregnancy. Geneva: WHO. 34432398

